# Retraction: Designing an intelligent monitoring system for corn seeding by machine vision and Genetic Algorithm-optimized Back Propagation algorithm under precision positioning

**DOI:** 10.1371/journal.pone.0294884

**Published:** 2023-11-21

**Authors:** The PLOS ONE Editors

The *PLOS ONE* Editors retract this article [1] because it was identified as one of a series of submissions for which we have concerns about peer review integrity and similarities across articles. These concerns call into question the validity and provenance of the reported results. We regret that the issues were not identified prior to the article’s publication.

All authors did not agree with the retraction.
